# Pseudoseptic Arthritis: A Case Series and Review of the Literature

**DOI:** 10.1155/2011/942023

**Published:** 2011-10-13

**Authors:** Brian P. Oppermann, Jonida K. Cote, Stephanie J. Morris, Thomas Harrington

**Affiliations:** ^1^Department of Rheumatology, Geisinger Health System, 200 Scenery Drive, Danville, PA 16801, USA; ^2^Division of Rheumatology, Rose Tree Medical Associates, Media, PA 19063, USA

## Abstract

*Purpose*. Pseudoseptic arthritis is an acute inflammatory monoarthritis with a sterile synovial gram stain and culture. Pseudoseptic arthritis has been previously described in the literature in a variety of settings including rheumatoid arthritis and microcrystalline disease. Despite pseudoseptic arthritis being a described entity, there is little published data on this topic with no published reports since 1992. *Methods*. This paper was a retrospective chart review over a 20-year period that identified all rheumatology inpatient consultations at our tertiary rural hospital for pseudoseptic arthritis. *Results*. We identified 10 patients with pseudoseptic arthritis and presented 5 of those cases in this paper. Majority of these patients had known autoimmune inflammatory arthritis or microcrystalline inflammatory arthritis. *Conclusion*. Pseudoseptic arthritis is a syndrome that should be in the differential diagnosis with patients with long standing inflammatory condition who present with an acute monoarthritis with no known bacterial source for septic arthritis.

## 1. Introduction

Pseudoseptic arthritis is an inflammatory arthritis which cannot be differentiated from septic arthritis on the basis of history, clinical presentation, or serum lab values [1]; therefore it often is confused with septic arthritis. Synovial fluid analysis is required to rule out septic arthritis which is a more serious condition. 

Many types of arthritis present similarly. Patients usually complain of an acute painful swelling of an erythematous and warm joint. Fever may be present. In pseudoseptic arthritis, the synovial fluid might look purulent but is sterile, gram stain shows no organism, and synovial fluid cultures are negative [2]. 

There are a few potential causes that lead to a diagnosis of pseudoseptic arthritis. Those include Behcet's disease, rheumatoid arthritis (RA), pseudogout, gout, reactive arthritis [3], acute lipoarthrosis in a systemic lupus erythematosus (SLE) pt [4], medications such as viscous injections with hyaluronic acid [5] or TNF-alpha blocker (etanercept) [6], trauma to the joint, intra-articular injection, or repeat injections [5]. These unique entities are all connected by a negative synovial fluid culture; however each has its own unique feature which distinguishes it from the other. We observed patients with rheumatoid arthritis, gout, prosthetic joint, renal transplant on immunosuppressive therapy and relapsing polychondritis. Other differential diagnosis beside septic arthritis are Lyme disease, and viral (reactive) arthritis [1]. 

In this paper, ten patients are reviewed that presented to a large tertiary care hospital with initial concern for septic arthritis that eventually were diagnosed with pseudoseptic syndrome. The patients reviewed had chronic diagnoses that include rheumatoid arthritis, gout, relapsing polychondritis and a patient with prosthetic joint replacement. Five of these patients are presented to illustrate the varying presentations and diagnostic studies for this syndrome. The demographics are listed in Table 1, and the diagnostic characteristics are shown in Table 2. 

## 2. Case 1

The patient, 65 y/o white male with long standing seropositive, erosive rheumatoid arthritis, and psoriasis, was initially admitted to the hospital secondary to erythroderma associated with his psoriasis. Subsequently, during his hospital stay he developed an acutely swollen, erythematous painful right shoulder. He was afebrile but was experiencing diaphoresis, tachycardia, and hypertension. Initially, his sedimentation rate (ESR) was 79, and WBC count was elevated at 26,000 with 82% segs. Joint exam revealed a warm, swollen right shoulder while other joints were consistent with quiescent RA. Joint aspiration yielded 15 mL's of cloudy fluid. Cell count was 262,000 with predominant PMNs. Gram stain, culture, and crystal analysis were negative. A presumptive diagnosis of septic arthritis was entertained, and he was placed on cefazolin for several days. In addition, he was prescribed rofecoxib 50 mg for 3, and prednisone was increased from 6 mg to 10 mg daily. Reaspiration the following day showed cell count of 130,000, and again cultures were negative. Symptoms improved over the next several days, and he was able to be discharged home with a diagnosis of pseudoseptic arthritis. 

He then presented 2 weeks later with a left lower lobe pneumonia. During this hospitalization, he developed a swollen, warm left shoulder. Aspiration yielded a cell count of 446,428 (PMN predominant) with negative gram stain. Again there was concern for a septic arthritis, and he was placed on antibiotic coverage with vancomycin. Due to the recent history of pseudoseptic arthritis of the right shoulder, his prednisone was increased to 20 mg with a slow taper, and rofecoxib was added. Cultures again were negative, antibiotics were discontinued and he was diagnosed with pseudoseptic left shoulder.

## 3. Case 2

The patient, 69 y/o male with long standing seropositive RA, presented to the emergency department with an acutely swollen left knee. He was on chronic immunosuppression consisting of daily low-dose prednisone and leflunomide (20 mg daily). Over a 24 hr period he developed a swollen, warm left knee and was unable to ambulate. Prior to presenting to the emergency department he had a temperature of 38 C. X-ray showed degenerative changes and joint aspiration yielded 40 mL's of cloudy fluid. Cell count was 63,390 (92% PMNs), and gram stain and cultures were negative. At the time of aspiration, knee was injected with methylprednisolone. He was covered with antibiotics until culture results. Within 24 hours, his left knee was significantly improved with minimal effusion, and he was able to ambulate without pain. He continued to be afebrile, antibiotics were discontinued, and he was diagnosed with pseudoseptic arthritis.

## 4. Case 3

Patient is a 55-year-old white male with multiple medical problems including hypertension, diabetes, chronic kidney disease, gout, COPD, obesity, sleep apnea who was admitted to the hospital by the ENT service for an elective endonasal septorhinoplasty. His postoperative course was complicated by acute respiratory distress syndrome secondary to aspiration pneumonia requiring intubation and admission to the adult intensive care unit. Few days later his status improved and was able to be transferred to a general medical floor for ongoing care. Four days into his medical course he was noted to have fevers (39.2°C) and a mild elevation of peripheral WBC count 11.36. Blood cultures, urine cultures were negative as well as lower extremity duplex imaging and VQ scan for a thrombosis and/or pulmonary embolus. He continued with low-grade fevers, and given recent aspiration pneumonia, was restarted on antibiotics. The following day he noted bilateral knee pain and swelling. His symptoms were so severe that he could no longer ambulate which lead to a concern for septic arthritis. There was a remote history of gout in the past with 2 episodes over 20 yrs but in the left foot. Aspiration of the right knee revealed 10 mL of cloudy serosanguinous synovial fluid. Cell count and differential revealed WBC of 115,000 with mainly neutrophils. Gram stain and culture were negative. Crystal analysis revealed negative birefringent monosodium urate crystals. Serum uric acid level was 10.1. It was determined that he had a pseudoseptic syndrome from gout and was treated with intra-articular corticosteroid injection. The following days his pain, swelling, and fever resolved.

## 5. Case 4

The patient is a 42-year-old white male who presented to the emergency room for evaluation of six-week duration of worsening severe left knee pain. His past medical history was significant for gout, hypertension, dyslipidemia, obesity, and reflux disease. He was diagnosed with gout two years prior to admission and experienced three to four attacks per year. He reported that he developed erythema, swelling, difficulty walking, and low-grade fevers. In the emergency room, he was found to have a temperature of 38.3°C, total WBC was 9.14, sedimentation rate was 15 and uric acid was 8.7. Arthrocentesis of his left knee revealed 45 mL of cloudy synovial fluid. Cell count and differential was 75,000 WBCs with 86% PMN's. Crystal analysis performed by the lab was negative, and gram stain revealed no organisms. Patient was admitted for concern of septic arthritis and was given a dose of ceftriaxone. The following day knee was again aspirated for 8 mL's of cloudy fluid. Light microscopy revealed bi-refringent needle-shaped intracellular uric acid crystals consistent with an acute gout flare. Cultures were negative. It was felt that his presentation was more consistent with a pseudoseptic arthritis from an underlying acute gout attack. His left knee was injected with methylprednisolone 40 mg and he was initiated on an oral prednisone taper for 12 days, antibiotics discontinued. Patient was seen one week later with complete resolution of his pain and he was restarted on his uric acid-lowering medication.

## 6. Case 5

A 68-year-old white female, with h/o breast cancer, bilateral total knee replacements, atrial fibrillation, CKI, presented with acute onset of right knee pain and swelling. She had a history of septic left knee one year prior to admission. She was placed on empiric ceftriaxone. Her right knee was tender to palpation, and an effusion was present. Her right knee was aspirated for 10 mL of bloody fluid, WBC 18, 300, no crystals present; gram stain and cultures were negative. X-ray of the right knee showed satisfactory alignment of the right knee replacement. Her knee was reaspirated for 23 mL of bloody fluid, WBC 44,000, predominately neutrophils, RBC 78,000. Her symptoms improved after arthrocentesis, and she was discharged home.

## 7. Discussion

Pseudoseptic arthritis is an inflammatory arthritis not due to a bacterial infection [3] and is a diagnosis of exclusion. This paper observed patients with pseudoseptic syndrome with varying diagnoses including rheumatoid arthritis, relapsing polychondritis, prosthetic joint, gout, and renal transplant patients on immunosuppressive therapy. Initially, all patients presented with typical features of septic arthritis due to suspected bacterial infection. Patients complained of intense pain, effusion, and erythema of a joint, most commonly knee. Fever and elevated inflammatory markers were present in some but not all of the cases. 

On arthrocentesis, synovial fluid was either cloudy, serosanguinous, or contained pus. In these cases, similar to the patients described by Mandell [7], there was intense synovial fluid leukocytosis, but synovial fluid gram stain and cultures were negative. Synovial fluid WBC count ranged from 18000 to greater than 400,000. In these gout patients, the other pertinent finding was negatively birefringent monosodium urate crystals. In all of these cases presented, despite intense leukocytosis, synovial fluid gram stain and culture were persistently negative. Initially patients were covered with antibiotics for suspected septic arthritis. Upon return of negative synovial fluid culture, antibiotics were discontinued and patients were treated with either oral or intra-articular steroids and sometimes NSAIDs. All patients improved within a couple of days following steroid treatment. They were discharged after an average of 6 days of hospitalization.

Before the diagnosis of pseudosepsis syndrome can be made, septic arthritis needs to be ruled out as it is most serious and potentially fatal disease [1]. A purulent joint should be considered septic until proven otherwise. According to Bradley and colleagues [8] pseudogout may mimic or mask septic arthritis. There was a concern that this may be true for the gout patients presented. Therefore, it is important to start antibiotic therapy on initial presentation until microbiological analysis of synovial culture is complete. If organisms are not isolated and the culture is negative, then the antibiotic regimen may be discontinued [2]. This approach seems to be especially reasonable in severely ill patients. According to Call and colleagues [2] when there is a synovial fluid leukocytosis with greater than 90% PMNs, antibiotics should be implemented, even though synovial fluid gram stain is negative. A decision of “not to treat” is easier if a patient has had previous episodes of culture-negative arthritis, is not in immunosuppressed state, does not have an identified port of entry, or has no concomitant infection [2]. In this way, the use of unnecessary antibiotic treatment and potential for bacterial resistance would be avoided. 

Pseudosepsis is not a self-limiting disease; instead it requires clinical intervention including arthrocentesis, intra-articular corticosteroids, and oral therapy with either non-steroid anti-inflammatory drugs or prednisone [5]. In our cases, patients improved tremendously after the appropriate therapy. The rapid resolution of symptoms is unlikely to be consistent with septic arthritis [7]. Recurrence is common. Similar to the patients reported by Mandell [7], none of the patients presented in this series suffered long-term sequences of morbidity or joint destruction.

In conclusion, pseudoseptic arthritis is a syndrome that should be kept in mind when faced with patients with long standing inflammatory condition who present with an acute monoarthritis with no known bacterial source for septic arthritis. In this paper, pseudoseptic arthritis was observed in a broad differential of disease including rheumatoid arthritis, gout, polychondritis, as well as after prosthetic joint replacement. This syndrome is an entity to be aware of, and truly the only way to differentiate pseudosepsis from septic arthritis is by negative synovial fluid culture and response to steroids [1]. 

## Figures and Tables

**Table 1:** Demographics.

Patient	Age	Sex	Main underlying diagnosis
1	65	M	RA, psoriasis
2	27	F	Renal transplant
3	51	M	Relapsing polychondritis
4	68	F	Prosthetic joint
5	88	F	RA
6	69	M	RA
7	56	M	RA
8	85	F	RA
9	76	M	Renal transplant
10	55	M	Gout

**Table 2:** Diagnostic characteristics.

Patient	Temperature °C	Joint involved	Peripheral WBC	ESR	Synovial fluid			
					WBC Cu/mm	Gram stain	Culture	Crystals
1 a	36.8	Shoulder—right	26.12	79	262,000	Neg	Neg	Neg
1 b	39.7	Shoulder—left	20.93	132	446,428	Neg	Neg	n/a
2	37.5	Knee—R	8.7	n/a	59,200	Neg	Neg	Neg
3	37.3	Knee—R	14.9	107	63,250	Neg	Neg	Neg
4 a	38	Knee—R	11.2	n/a	18,300	Neg	Neg	Neg
4 b	36	Knee—R	15.19	45	44,000	Neg	Neg	Neg
5	39.7	Knee—L	13.8	78	n/a	Neg	Neg	n/a
6	38.5	Knee—L	12	64	63,390	Neg	Neg	Neg
7	35.8	Hip—R	17.4	98	163,000	Neg	Neg	n/a
8	38.3	Hip—L	10.3	69	33,200	Neg	Neg	n/a
9	37.3	Elbow—R	12.94	29	138,400	Neg	Neg	+ uric acid
10	39.2	Knee—R	11.36	n/a	115,000	Neg	Neg	+ uric acid
